# Benchmarking of long-read correction methods

**DOI:** 10.1093/nargab/lqaa037

**Published:** 2020-05-25

**Authors:** Juliane C Dohm, Philipp Peters, Nancy Stralis-Pavese, Heinz Himmelbauer

**Affiliations:** Institute of Computational Biology, Department of Biotechnology, University of Life Sciences and Natural Resources, Vienna (BOKU), Muthgasse 18, 1190 Vienna, Austria

## Abstract

Third-generation sequencing technologies provided by Pacific Biosciences and Oxford Nanopore Technologies generate read lengths in the scale of kilobasepairs. However, these reads display high error rates, and correction steps are necessary to realize their great potential in genomics and transcriptomics. Here, we compare properties of PacBio and Nanopore data and assess correction methods by Canu, MARVEL and proovread in various combinations. We found total error rates of around 13% in the raw datasets. PacBio reads showed a high rate of insertions (around 8%) whereas Nanopore reads showed similar rates for substitutions, insertions and deletions of around 4% each. In data from both technologies the errors were uniformly distributed along reads apart from noisy 5′ ends, and homopolymers appeared among the most over-represented kmers relative to a reference. Consensus correction using read overlaps reduced error rates to about 1% when using Canu or MARVEL after patching. The lowest error rate in Nanopore data (0.45%) was achieved by applying proovread on MARVEL-patched data including Illumina short-reads, and the lowest error rate in PacBio data (0.42%) was the result of Canu correction with minimap2 alignment after patching. Our study provides valuable insights and benchmarks regarding long-read data and correction methods.

## INTRODUCTION

Sequencing technologies have developed toward high-throughput generation of long sequencing reads at the kilobasepair (kbp) scale using single molecules as templates. For *de novo* genome assembly and gene prediction such data are of great value. Long-read data allow to obtain improved contiguity as compared to short-read assemblies (1), facilitate the detection of structural variants between genomes (2,3) and can be used to generate full-length transcript sequences (4,5). The two most prominent platform providers are Pacific Biosciences (PacBio) and Oxford Nanopore Technologies (ONT) (6,7). The generated sequencing reads achieve peak lengths of about 10–20 kbp (PacBio and ONT) (8,9) and a maximum length of 2272 kbp (ONT) (10). However, such reads exhibit high error rates and need to be corrected before *de novo* assembly. Elevated error rates are expected whenever a single molecule is sequenced, in contrast to highly parallel short-read sequencing where local clusters of the same molecule are sequenced simultaneously, compensating for individual errors or out-of-phase templates and achieving very low error rates (11,12).

On a PacBio Single Molecule Real-Time sequencing device a single DNA molecule is immobilized in a zero-mode waveguide. The four nucleotides are added, each labeled with a differently colored fluorophore. When a base is incorporated by a DNA polymerase, the emitted light signal is detected, the corresponding base is called, and the fluorophore is cleaved off. In an ONT flowcell, a motor protein drags a DNA molecule through a nanopore. The current in the pore is measured during this process and bases are called according to the current profile. The R9.4 pore provides a signal for six nucleotides (nt) at a time, of which the central bases in the middle of the pore contribute the most.

The quality of reads and their error rates are monitored throughout the sequencing and base calling process and checked afterwards. Tools for quality control are for example the nanopore built-in MinKNOW, or fastQC (13), providing metrics such as per-base quality, distributions of read length, and counts of over-represented k-mers. With a reference sequence available, NanoOK (14) can assess the number of substitutions, insertions, and deletions. In the current state, long reads from these platforms display error rates of approximately 15% (15,16), hence have to be corrected during the assembly process in order to join them accurately into longer contigs.

The main approach for correction is the consensus correction either with a subset of the long-read data or with additional short reads. The first approach is applied in the correction step of the Canu assembler based on all-versus-all read overlaps (17). Also the tool MARVEL (18) uses this approach, but there is a preceding correction step that performs ‘patching’ of reads. The process of patching is supposed to repair large-scale errors (such as highly erroneous regions, missed adapters, or missing sequence information) based on comparisons with other reads from the dataset. The idea is that splitting of reads at erroneous regions will be prevented and long continuous reads remain intact to allow the generation of longer contigs during the assembly process. The second approach uses additional high-quality short-read data (e.g. generated by Illumina sequencing) in order to correct the long reads based on short-read mapping, as applied by nanocorr (19) or proovread (20).

For consensus correction by overlapping of long reads the device-dependent differences in the error distribution have to be taken into account. PacBio sequencing is expected to produce reads that have errors uniformly distributed across the read (6). ONT sequencing results are especially error prone in homopolymeric regions, mainly due to the design of the nanopore. Furthermore, the accuracy of ONT sequencing depends on the GC content (19). These differences are often already accounted for, e.g. Canu allows to set parameters to indicate from which sequencing platform the reads originate. Still, different long-read alignment methods deal better or worse with data from different platforms (21), potentially introducing biases (22). This may affect the results when assessing the quality of assembled contigs by aligning them to a reference (e.g. using mummer (23)) or searching for conserved genes (e.g. using BUSCO (24)).

Read correction is highly dependent on the applied methods and their specific parameters and is under constant development, since both the sequencing technologies and the alignment methods are evolving. Therefore it is important to assess the read quality and the error rates not only in raw reads, but also in corrected reads before they get assembled.

Here, we compare the correction steps implemented in two long-read assembly programs (Canu, MARVEL) and a correction procedure using additional short-read data (proovread). Our input data were generated from *Escherichia coli* using PacBio, Oxford Nanopore, and Illumina technologies. We compare error rates and k-mer occurrences in raw data and corrected data and determine changes in read lengths, read numbers, and maximum match lengths. We provide conclusions how to combine the tested correction steps in order to achieve lowest error rates.

## MATERIALS AND METHODS

### Input data

PacBio long-read data (RS II P6C4 chemistry) and Illumina paired-end data (2 × 100 nt, GA IIx instrument) from the *E. coli* strain K12 substrain MG1655 were obtained from public resources (Illumina data: NCBI SRA accession number ERX008638; PacBio data: https://github.com/PacificBiosciences/DevNet/wiki/E.-coli-Bacterial-Assembly accessed 6 September 2018).

For *E. coli* strain K12 substrain DH5α we generated Illumina paired-end data (2 × 125 nt, HiSeq 2500 instrument) as well as long-read data using the Oxford Nanopore Technology (ONT) with its MinKNOW base caller and the SQK-RAD004 sequencing kit. We ran the MinION sequencing instrument using an R9.4 flowcell (FLO-MIN106) for 48 hours. Base calling was performed using MinKNOW version 1.13.1. The raw data have been deposited in the NCBI Sequencing Read Archive under BioProject PRJNA610591, BioSample SAMN14306692.

The total coverage of 784× ONT data and 161× PacBio data were aligned to the *E. coli* references of DH5α (NCBI nucleotide accession number CP017100) and MG1655 (NCBI nucleotide accession number NC_000913.3), respectively, using minimap2 (25) with parameters –secondary = no -L –MD -x map-ont (or -x map-pb, respectively). For each dataset the matching fraction of reads was downsampled to 50× coverage for further analysis using seqtk sample with -s 23 as seed (https://github.com/lh3/seqtk/ accessed 2016 Feb 22). Only reads larger than 200 bp were considered. Illumina data of both strains were trimmed for adapter sequence using trimmomatic v. 0.35 (26) and the setting ILLUMINACLIP:TruSeq3-PE.fa:2:30:10, aligned to the respective *E. coli* reference using bowtie (27) with parameters –nomaqround -v 3 –best, and downsampled to 320× coverage of matching reads for each sample using seqtk.

The alignment of ONT reads indicated that our version of *E. coli* K12 substrain DH5α differed from the public reference labeled as ‘NEB 5-α, a derivate of K12 DH5α’ by a deletion of ∼1300 bp. To avoid biases regarding the error rates we removed ONT reads that were affected by this deletion before downsampling. For K12 substrain MG1655 no such deletion was found compared to the reference.

### K-mer counting

The k-mers in the reference genomes were counted using jellyfish (28) version 2.2.10 using both forward and reverse strands taking into account the circular nature of the *E. coli* genome (i.e. k-mers bridging the end and start of the linear fasta-string were counted, too). Raw, patched and corrected reads were treated as linear sequences. Since nanopore sequencing on the MinION device provides a signal for 6 nt at a time, we focused on the analysis of k-mers of size *k* = 6.

Sequence logos were generated at https://weblogo.berkeley.edu/logo.cgi (accessed 6 February 2020) with the option ‘frequency plot’. The input was the list of the 30 most over-represented six-mers in ONT or PacBio data, respectively, from which homopolymers were removed before logo generation. In the case of PacBio data, all four homopolymers were among the top 30 over-represented six-mers, whereas in case of the ONT data polyC was not contained. Thus, the sequence logos were generated based on 26 six-mers (PacBio data) and 27 six-mers (ONT data), respectively.

### Alignment methods

Alignments were performed using NanoOK (14) (version 1.34), calling the methods graphmap (version 0.5.2), last (version lastal 921) and minimap2 (version 2.12-r827). For minimap2, the sequencing platform was provided as parameter (-x map-ont, -x map-pb) otherwise default parameters were chosen, leading to slight differences compared to the initial selection of reads. For graphmap, the parameter for the circular nature of the *E. coli* genome was set (–circular) and –alg anchor. As a fourth method blasr (29) (version 5.3) was used with parameters –bestn 1 –hitPolicy randombest –randomSeed 42 –sam –header –printSAMQV. The error rate statistics were extracted from NanoOK output for graphmap, last and minimap2 alignments; both blasr output analysis and the calculation of error distribution was done by custom scripts written in python 2.7.

### Correction methods

For patching and correction of ONT and PacBio reads, MARVEL (18) (version 1.0, commit 1f693baf8420c2121cc40d18ed2088c6e81a713b) was used. Parameters for PacBio and ONT data were applied on *E. coli* data as provided in the program's example files. The differences between the treatment of ONT and PacBio data consisted of higher quality thresholds for quality trimming and fixing of the ONT data. The patched reads before further correction were analyzed separately (provided by MARVEL as intermediate files).

The correction step of Canu (17) (version 1.6) was applied both to the raw and patched reads of both datasets. Canu provides a parameter setting for specific sequencing data (nanopore-raw/pacbio-raw). Correction parameters were set as advised by the manual (correctedErrorRate = 0.105), which affect the reads in the assembly steps after the initial correction. As overlapper tools both the in-built MHAP and minimap2 were applied.

As hybrid correction method, proovread (20) (version 2.13.11) was applied on raw and patched reads, using the Illumina short-read datasets of DH5α or MG1655, respectively.

All programs and analyses were run on a high-performance Linux cluster using machines with 128 GB of RAM and 24–32 cores.

## RESULTS

### Error rates of raw reads

**Figure 1.**
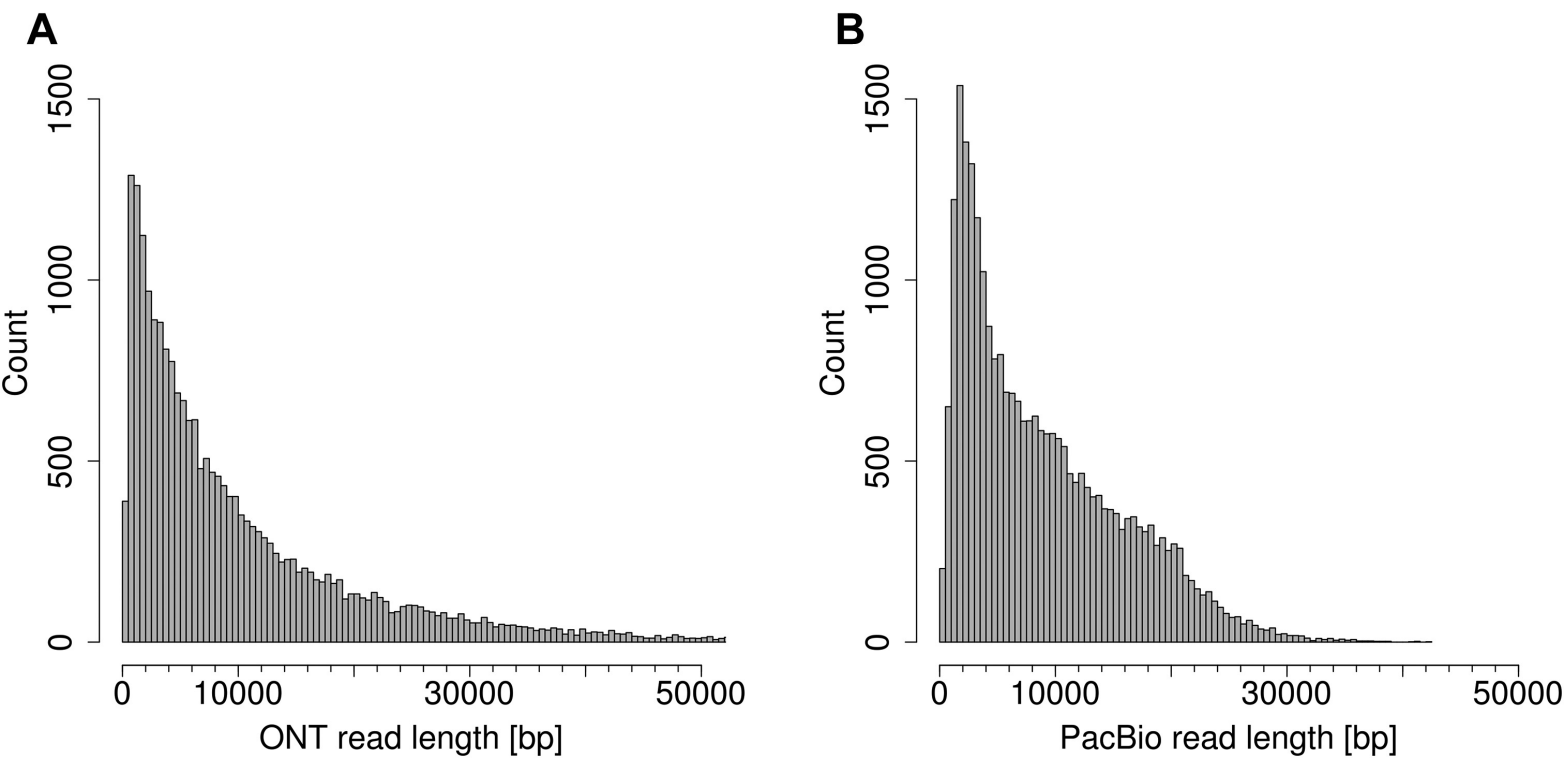
Read length distributions up to 50 kbp for ONT (**A**) and PacBio (**B**). The longest ONT read was 136 kbp.

The analyses were performed using long-read and short-read data from *E. coli* strain K12 substrains DH5α and MG1655. We generated long-read data using the Oxford Nanopore technology (ONT) and Illumina short-read data for DH5α and obtained PacBio data and Illumina data for MG1655 from public sources (Figure 1). The ONT data generated comprised 346 489 reads (3.60 Gbp) corresponding to ∼784-fold coverage of the *E. coli* genome, and the average read length was 10 377 bp with a maximum of 136 180 bp. After alignment of the four datasets to the *E. coli* references of either DH5α or MG1655, respectively, only 50-fold coverage of long-read data and 320-fold coverage of short-read data of the matching fraction for each reference were kept for further analysis (Table 1).

**Table 1.** Initial datasets after downsampling (matching reads only)

Technology	*E. coli* strain	Number of reads	Coverage	Mean read length [bp]	Longest read [bp]
ONT (R9.4, 1D)	DH5α	22 000	50×	10 508	136 180
Illumina HiSeq	DH5α	11 763 308	320×	125	125
PacBio (RS II P6C4)	MG1655	26 300	50×	8899	42 279
Illumina GAIIx	MG1655	14 750 994	320×	101	102

We estimated the rates of substitutions, insertions, and deletions in the raw reads based on mapping results of four different alignment methods. The tools blasr (29), graphmap (30), last (31) and minimap2 (25) were used to align ONT and PacBio reads (50-fold coverage each) against the respective reference genomes. The alignments showed similar total error rates for ONT (average 12.56%, standard deviation 0.34) and PacBio reads (average 12.88%, standard deviation 0.76), respectively, whereas the rate of the specific error types differed across alignment tools as well as across sequencing methods (Table 2). PacBio data contained lower rates of substitution errors but much higher rates of insertions than ONT data, and deletions occurred slightly less frequently in PacBio reads than in ONT reads. The highest error rates were observed when using graphmap, the lowest error rates resulted from using last for ONT reads and from using minimap2 for PacBio reads. For both datasets, last showed higher rates of substitutions and lower rates of insertions/deletions (indels) than the other alignment programs; blasr, in contrast, resulted in lower rates of substitutions and a higher rate of indels.

**Table 2.** Error rates as obtained from raw read alignments against *E. coli* K12 DH5α (ONT reads) and K12 MG1655 (PacBio reads)

Dataset	Alignment tool	Aligned reads [%]	Substi-tutions [%]	Insertions [%]	Deletions [%]	Total error [%]	Longest perfect match [bp]	Longest alignment [bp]
ONT	blasr	99.76	3.50	4.01	4.94	12.45	369	99 183
ONT	graphmap	99.72	4.38	3.94	4.76	13.08	373	142 108
ONT	last	100.00	5.12	3.23	4.00	12.35	373	98 422
ONT	minimap2	99.83	4.33	3.59	4.47	12.39	371	98 780
**ONT**	**average**	**99.83**	**4.33**	**3.69**	**4.54**	**12.56**	**372**	**109 623**
PacBio	blasr	98.35	1.07	8.33	3.43	12.83	191	42 971
PacBio	graphmap	98.18	1.81	8.82	3.32	13.95	189	49 318
PacBio	last	100.00	2.07	7.40	2.89	12.36	192	42 468
PacBio	minimap2	98.01	1.75	7.60	2.98	12.33	189	42 737
**PacBio**	**average**	**98.89**	**1.68**	**8.04**	**3.16**	**12.88**	**190**	**44 373**

Despite similar overall error rates, ONT data had better metrics than PacBio data regarding the read match lengths when mapping against the reference. The longest perfectly matching sequence and the longest alignment were both about two times longer in ONT reads than in PacBio reads; the latter can be attributed to the lack of reads larger than 42 kbp in PacBio data.

The longest of all alignments for the ONT dataset was achieved with graphmap, aligning the longest read (length = 136 180 bp) with a high rate of deletions to a region of 142 108 bp. The other alignment tools aligned only 80 000 bases of this read and introduced soft-clipping at one end. The second longest alignment of graphmap was the longest of the three other tools using the second longest read (length = 95 545 bp) of the dataset. All alignments exceeded the length of the read by 3.0–3.8% (Table 2).

For PacBio data the alignments of blasr, last and minimap2 did not exceed the length of the reads as much as in ONT data. The longest read in the dataset (length = 42 279 bp) was stretched by 700 bp (+1.6%) in the longest blasr alignment. Again, graphmap provided the longest alignment and stretched the read by 7000 bp (+16.6%). The read was mapped to the same region in the reference sequence by all alignment methods.

**Figure 2.**
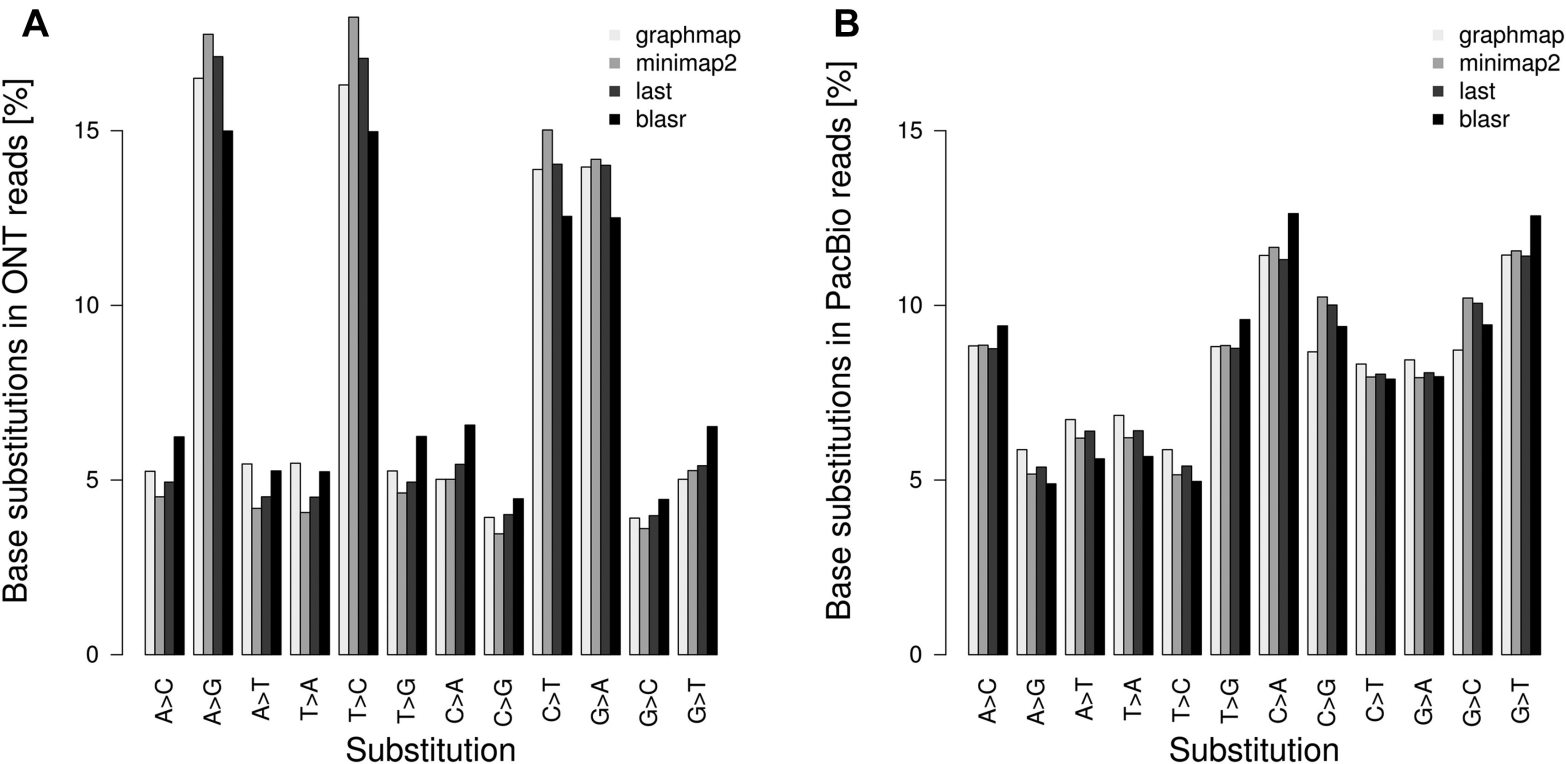
Base substitution rates of aligned reads by four alignment methods for ONT (**A**) and PacBio (**B**) sequencing data. Rates were determined by NanoOK and in-house scripts.

Assessing substitution errors in the read data is especially important for variant calling. We compared substitution rates for transversions and transitions in ONT and PacBio reads based on alignments of the four different alignment tools (Figure 2). While ONT seemed to have a few top candidates for substitution errors, PacBio substitutions appeared to be more balanced, but not completely random, as assumed previously (6). In ONT reads, substitution rates for the transitions A↔G and C↔T were clearly elevated. In PacBio reads, substitution rates for the transversions A↔C and G↔T were slightly elevated. All the tested alignment tools confirmed this tendency.

**Figure 3.**
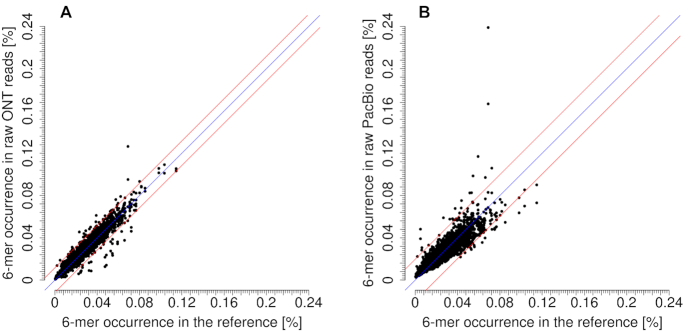
Comparison of occurrences of six-mers in the reference genomes and the raw read datasets of ONT (**A**) and PacBio (**B**). The diagonal blue line stands for perfect representation. The two red lines show the 3-fold standard deviation (ONT stddev = 0.0039, PacBio stddev = 0.0066).

**Figure 4.**
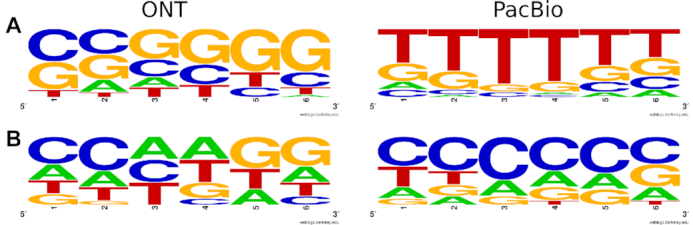
Sequence logos of the top 30 over-represented (**A**) six-mers (excluding homopolymers) and top 30 under-represented (**B**) six-mers in the raw read datasets of ONT (left) and PacBio (right) compared to the reference.

In order to further asses the resulting sequence accuracy of the specific sequencing technique, k-mer occurrences in the reference genomes were counted and compared to the respective occurrences in the raw read datasets (Figure 3 and Supplementary Figure S1). Nanopore's known problems with sequencing homopolymers became apparent for the homopolymers of ‘A’ and ‘T’, whereas ‘G’-homopolymers were less strongly affected and ‘C’-homopolymers did not even appear in the list of 30 most over-represented k-mers (Table 3 and Supplementary Table S1). PacBio data were affected by homopolymer over-representation as well, in fact for all four kinds of homopolymers (Table 3). T-rich six-mers were found to be over-represented in PacBio data but were among the under-represented six-mers in ONT data; in contrast, G and C were more often in over-represented six-mers of ONT data (Figure 4 and Supplementary Table S1).

**Table 3.** The ten most over- and under-represented six-mers in the raw read datasets compared to the reference

	ONT		PacBio	
	6-mer	difference in rate	6-mer	difference in rate
**over-represented**	TTTTTT	0.0573	TTTTTT	0.1699
	AAAAAA	0.0260	AAAAAA	0.0977
	GCACGG	0.0224	GTTTTT	0.0575
	CGGGCG	0.0221	TTTTTG	0.0439
	CGGCGG	0.0186	GGGGGG	0.0432
	CACGGC	0.0174	CCCCCC	0.0341
	CAGCGG	0.0169	TTTTTC	0.0335
	GGGCGG	0.0166	TTTTGG	0.0323
	CGGTGG	0.0164	TGTTTT	0.0315
	CCGGGC	0.0164	GGTTTT	0.0309
**under-represented**	GCCTGG	−0.0347	CGCCAG	−0.0418
	CCTGGC	−0.0345	CCAGCG	−0.0346
	CAAAAA	−0.0311	GCCAGC	−0.0334
	AAAAAG	−0.0292	CCAGCA	−0.0305
	AAAAAT	−0.0282	CACCAG	−0.0278
	GAAAAA	−0.0260	CCGCCA	−0.0272
	CCAGGC	−0.0258	TGCCAG	−0.0263
	ACCTGG	−0.0257	TCGCCA	−0.0263
	TAAAAA	−0.0252	ACCAGC	−0.0263
	GCCAGG	−0.0250	ATCGCC	−0.0261

Under-represented six-mers of PacBio data were C-rich, and there was a tendency to contain two C-nucleotides next to each other (Figure 4 and Supplementary Table S1). In ONT data under-represented six-mers were often composed of 5 nt A or T plus one of the respective other three bases (Supplementary Table S1).

In general, the standard deviation (stddev) for all six-mers with respect to their occurrence in the reference was lower for the ONT than for the PacBio dataset (ONT: stddev = 0.0039, PacBio: stddev = 0.0066).

For k greater than 6, k-mers occurring in low frequencies in the reference tended to be over-represented in both raw read datasets, whereas k-mers occurring in high frequencies in the reference tended to be under-represented in the raw read datasets (Supplementary Figure S1).

### Error distribution and correction by ‘patching’

During the ‘patching’ process of the tool MARVEL (18), reads are compared to each other and to the adapter sequence, and highly erroneous regions are substituted with information from other reads of the dataset. The aim is to obtain uniform quality across reads to prevent splitting of the long reads into smaller pieces, which would occur when cutting out low-quality regions.

We applied the patching step of MARVEL on the 50-fold coverage datasets of ONT and PacBio reads and compared patched and unpatched read data. Apart from a slight decrease of overall error rates (Table 4) the patching resulted in differences in read lengths and number of reads. The longest patched read in the ONT data was longer than the longest raw read (raw: 136 180 bp, patched: 136 895 bp). In PacBio data, the longest patched read was shorter than the longest raw read (raw: 42 279 bp, patched: 41 257 bp). After patching, the number of reads longer than 20 kbp increased in the ONT dataset from 3388 to 3417, and decreased from 2173 to 2033 in the PacBio dataset. The patching process removed ∼4000 reads of lengths <2000 bp from each of the datasets, i.e. all reads shorter than 2000 bp from the ONT data and 98% of reads shorter than 2000 bp from the PacBio data.

**Table 4.** Error rates after alignment of patched and corrected reads against the respective reference genome using blasr

	unpatched				MARVEL patched				
Consensus correction	None	Canu MHAP	Canu minimap2	Proovread	None	MARVEL	Canu MHAP	Canu minimap2	Proovread
	**ONT**				**ONT**				
**Substitution %**	3.50	0.20	0.19	0.25	3.19	0.21	0.17	0.16	0.10
**Insertion %**	4.01	0.19	0.18	0.22	3.81	0.26	0.17	0.16	0.10
**Deletion %**	4.94	1.65	1.67	0.78	4.21	0.89	1.25	1.21	0.25
**Total error %**	**12.45**	**2.04**	**2.04**	**1.25**	**11.21**	**1.36**	**1.59**	**1.53**	**0.45**
**Longest perfect match (bp)**	369	1694	1845	84 881	369	1275	1819	1833	92 302
	**PacBio**				**PacBio**				
**Substitution %**	1.07	0.08	0.07	0.15	0.93	0.05	0.08	0.07	0.08
**Insertion %**	8.33	0.30	0.22	1.19	7.22	0.35	0.28	0.17	0.55
**Deletion %**	3.43	0.26	0.24	0.23	3.02	0.21	0.26	0.18	0.09
**Total error %**	**12.83**	**0.64**	**0.53**	**1.57**	**11.15**	**0.61**	**0.62**	**0.42**	**0.72**
**Longest perfect match (bp)**	191	14 682	14 756	33 315	191	4398	18 450	12 712	39 811

The MARVEL patching only slightly changed the occurrences of non-homopolymer six-mers in ONT data (stddev = 0.0003 between patched reads and raw reads) and in PacBio data (stddev = 0.001) (Supplementary Figure S2). However, the number of over-represented homopolymers decreased due to patching.

**Figure 5.**
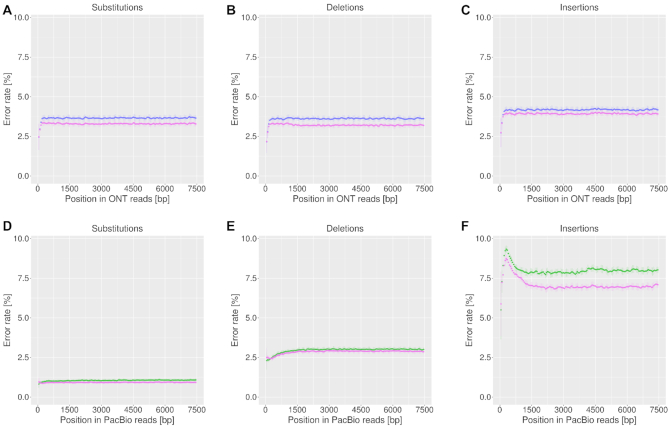
Error distribution along raw PacBio reads (green) and raw ONT reads (purple) for substitutions (A, D), deletions (B, E) and insertions (C, F). Error rates slightly decreased after MARVEL patching (pink). The error rates were determined in sliding windows of length 1 kbp with 0.5 kbp overlap for positions 1–7500 of reads longer than 7500 bp. Error bars show the standard deviation per window.

We assessed whether there was a bias in the distribution of errors along the reads and compared the error distribution after patching. Considering positions 1–7500 of reads longer than 7500 bp we found for both types of sequencing data that substitutions, insertions and deletions were rather uniformly distributed apart from noisy 5′ ends (Figure 5). Error rates in ONT data ranged around 4% (substitutions, deletions) and 4.5% (insertions). The larger differences between error types in PacBio became obvious in the error distributions with substitutions ranging around 1%, deletions around 3% and insertions around 8%. Additionally, insertions in PacBio data showed a high error peak at the beginning of reads. After MARVEL patching the error rates only slightly decreased along the reads (Figure 5).

### Consensus correction

**Figure 6.**
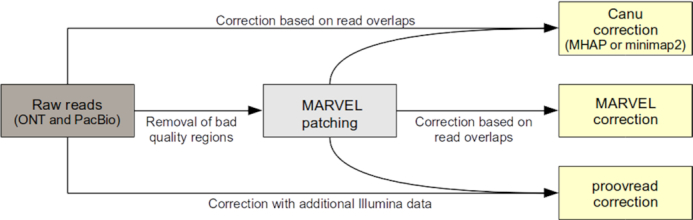
Workflow of the applied correction steps.

**Figure 7.**
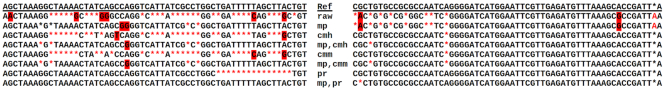
Alignments of ONT reads against two example regions of the *Escherichia coli* DH5α reference sequence as raw reads and after applying correction steps. Red asterisks indicate deletions, red characters indicate insertions, characters highlighted in red indicate mismatches. Left side: positions 1816110–1816169, right side: positions 1813540–1813599. Ref: reference, raw: raw read, mp: MARVEL patched, cmh: Canu MHAP, cmm: Canu minimap2, pr: proovread.

The main step to improve the quality of long reads is based on read overlapping to generate corrected consensus sequences. Since the patching step of MARVEL affects only particular regions of the reads we tested both unpatched and patched read datasets as input for consensus correction (Figure 6). Three consensus correction methods were compared: Canu (17), MARVEL and proovread (20) (Figure 7).

Canu and MARVEL perform a consensus correction using exclusively the dataset of long reads, whereas proovread makes use of complementary Illumina sequencing data. In contrast to polishing tools like Quiver/Arrow (https://github.com/PacificBiosciences/GenomicConsensus accessed 30 October 2019) or Pilon (32) that are applied on assembled contigs, proovread uses Illumina data to match them against the unassembled raw reads.

The two long-read-only correction methods Canu and MARVEL decreased the total error rate of ONT reads on average from 12.45 to 1.71% and the error rate of PacBio reads from 12.83 to 0.56%, respectively (Table 4). The Canu parameter for the origin of the data was set (-pacbio-raw, -nanopore-raw) and seemed to be better adapted to PacBio data than to ONT data. The higher remaining error rate in corrected ONT reads was mostly due to deletions, which could only be decreased to 1.33% on average, in contrast to a decreased deletion rate of 0.23% in corrected PacBio reads. The two different alignment programs used by Canu internally, MHAP (33) and minimap2 (25), resulted in similar results whereby the consensus correction based on minimap2 achieved slightly lower error rates than the correction based on MHAP alignments (up to 0.2% for patched PacBio reads, see Table 4).

Usage of additional Illumina sequencing data with proovread correction resulted in error rates of 0.85% for ONT and 1.15% for PacBio, respectively. Thus, interestingly, for ONT data the error rates after short-read correction were lower than using long-reads only, but for PacBio data the error rates were higher after short-read correction than after long-read correction. Short-read data with proovread achieved the lowest error rates for deletions in ONT data as compared to Canu and MARVEL; insertion rates in PacBio data remained the highest after short-read correction compared to long-read correction (Table 4).

**Figure 8.**
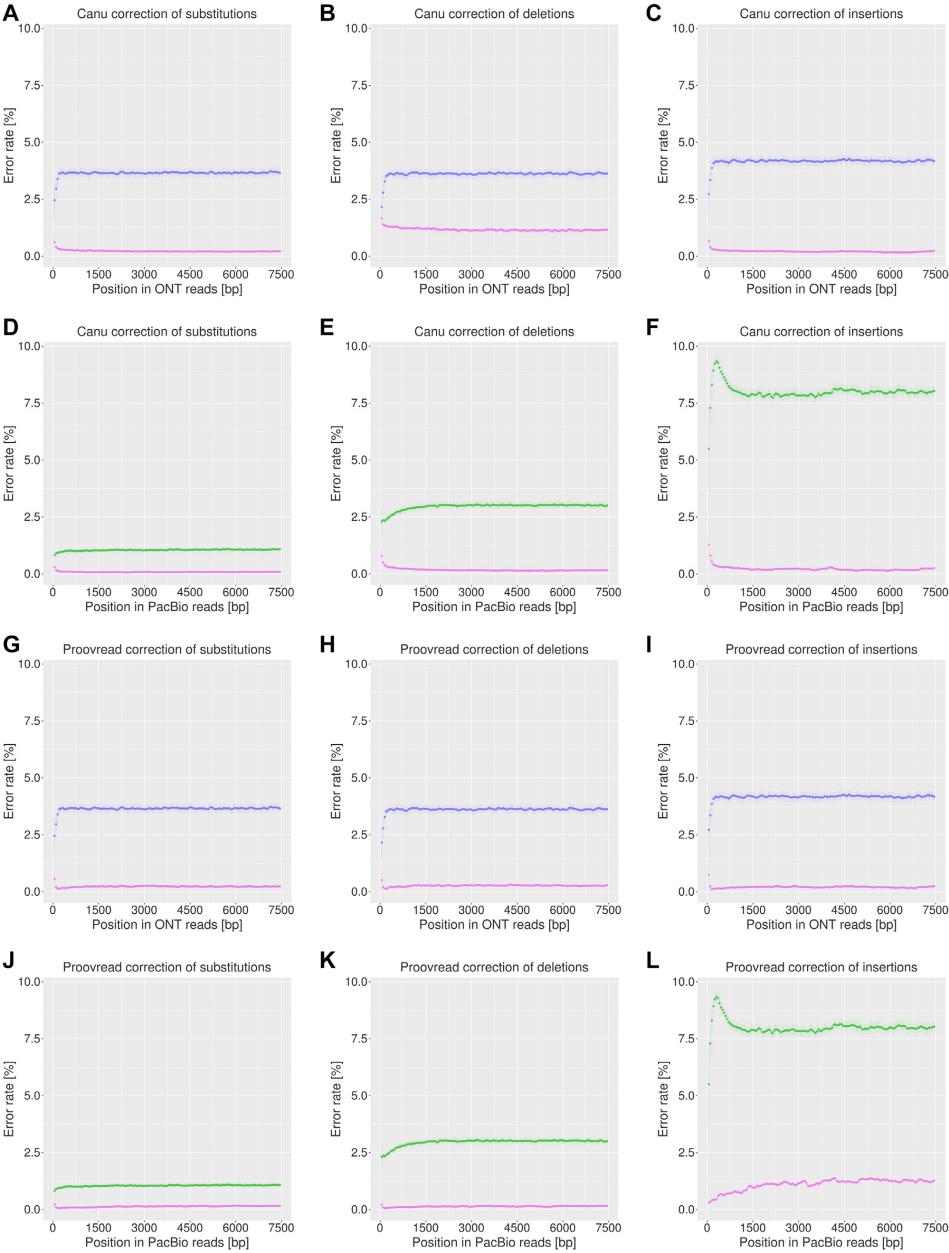
Error distributions along unpatched PacBio and ONT reads before (green and purple, respectively) and after (pink) consensus correction by Canu (**A**–**F**) or proovread (**G**–**L**) for substitutions, deletions and insertions. Reads were analyzed as in Figure 5.

The error rates along the reads (unpatched raw input data) after consensus correction by Canu and proovread were rather uniformly distributed except for the proovread correction of PacBio insertion errors which showed stronger variation (Figure 8).

The length of the longest perfect match increased drastically after short-read correction. The PacBio longest perfect match of raw reads (191 bp) increased after long-read correction to a length between 4 and 18 kbp depending on the correction method, but short-read correction achieved longest perfect matches of 33 to 40 kbp. The difference was even more pronounced for ONT data where the longest perfect raw-data match of 369 bp was increased on average to 1693 bp (range 1275–1845 bp) after long-read correction and to 85 kbp (unpatched ONT input) and 92 kbp (patched ONT input), respectively, after short-read correction.

After MARVEL correction we observed an increase in average read lengths and decreased read numbers. The MARVEL correction starts with concatenating the reads based on sufficiently supported overlaps. This generates a set of extended reads, and the number of corrected reads is much smaller than the number of initial input reads. For ONT, the number of 17 934 patched reads was reduced by MARVEL to 110 concatenated reads, containing in total 6 430 965 bases. The mean length of the reads increased from 10 508 to 58 463 bp. For PacBio, the number of 22 586 patched reads was reduced to 285 concatenated reads (in total 6 889 188 bp), and the mean length of the reads increased from 8899 to 24 172 bp. The sets of concatenated reads showed lower error rates than the Canu-corrected reads for both sequencing technologies, but a shorter perfect match length (Table 4).

In general, patched read data as input resulted in lower error rates than unpatched read data, with all correction methods. This was observed for ONT data as well as for PacBio data.

**Figure 9.**
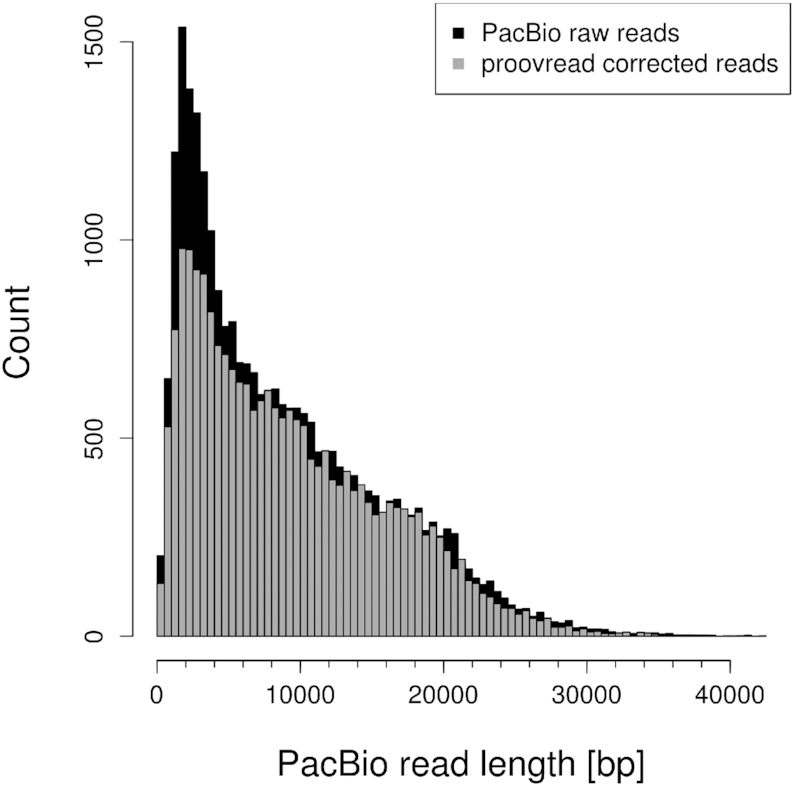
Read length distributions for raw PacBio reads and after proovread correction.

In the patching phase, MARVEL removed most reads shorter than 2000 bp. Further reads were removed from the datasets during correction. Canu removed most raw reads of length <7500 bp from ONT data and reads <4500 bp from PacBio data. From patched reads, Canu removed reads <8000 bp from ONT data and reads <5500 bp from PacBio data, respectively. During proovread correction reads were only removed from PacBio raw reads, and most of these removed reads had a length below 10 kbp (Figure 9). For ONT and patched reads, no reads were removed by proovread.

### K-mer occurrences after correction

**Figure 10.**
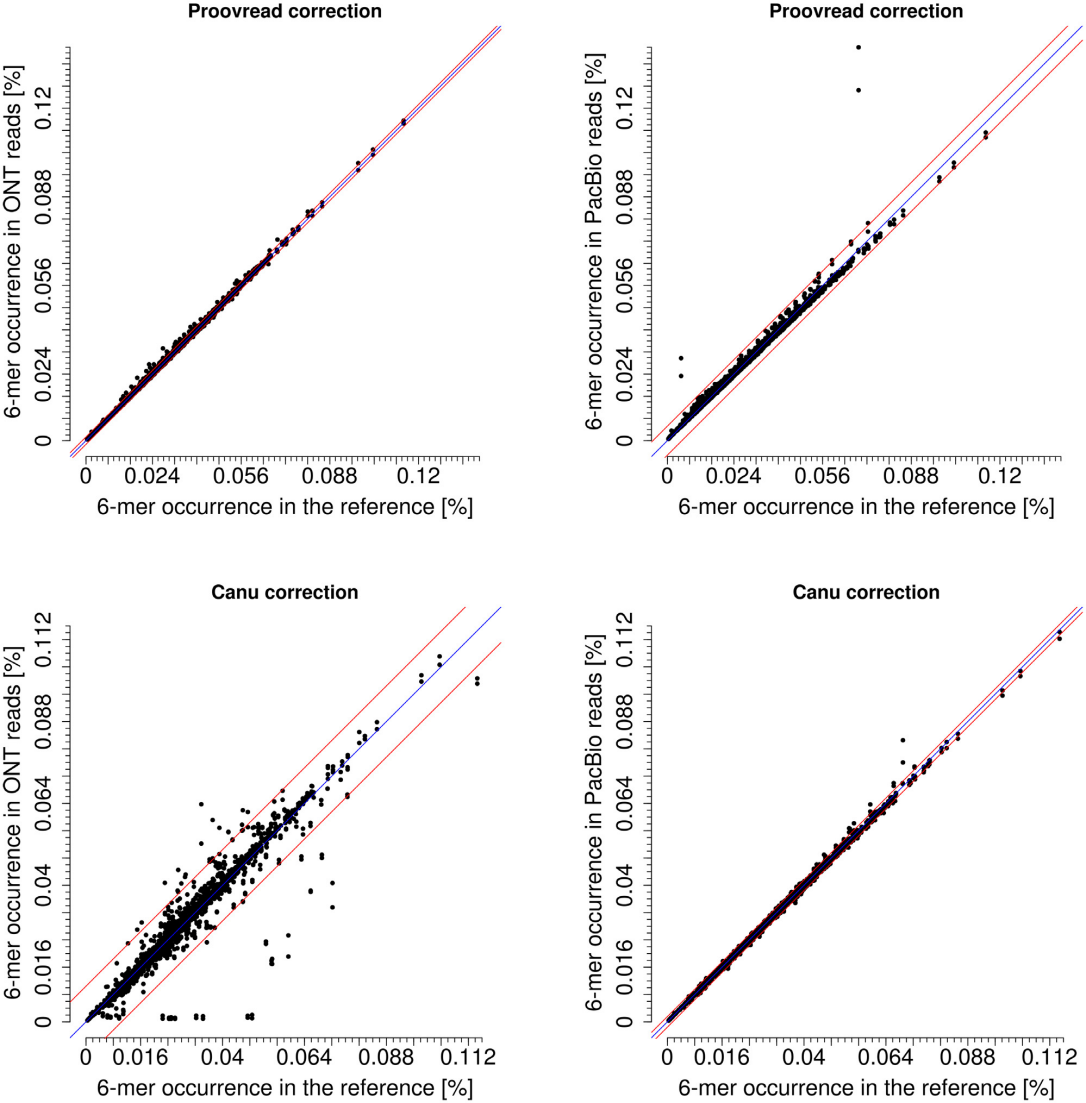
Frequencies of six-mers after correction by proovread and Canu (MHAP), respectively, in ONT reads (left) and PacBio reads (right) compared to the reference. Unpatched input reads were used. The diagonal blue line stands for perfect representation. The two red lines indicate the 3-fold standard deviation (proovread: ONT stddev = 0.0004, PacBio stddev = 0.0017, Canu: ONT stddev = 0.0035, PacBio stddev = 0.0005).

**Figure 11.**
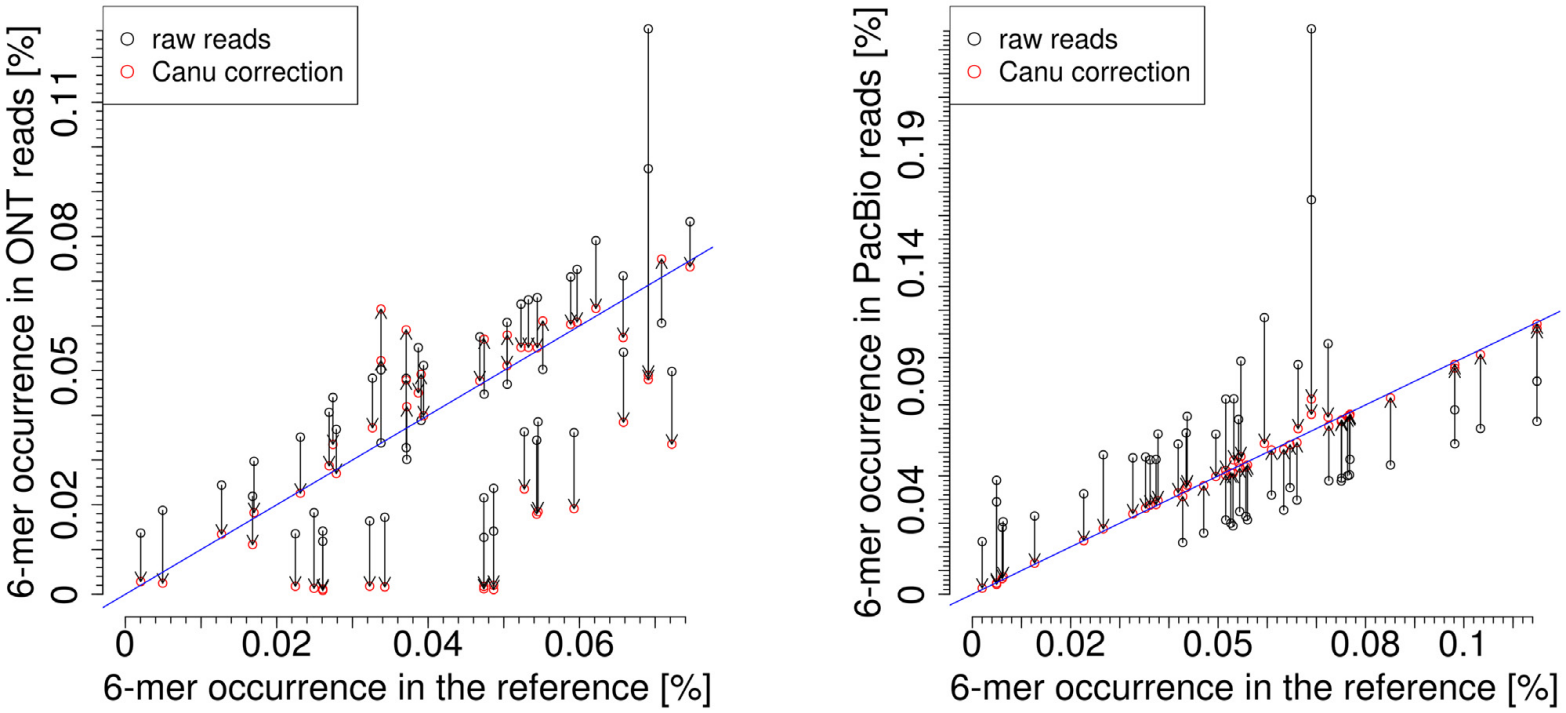
Change in frequencies of six-mers when applying the Canu (MHAP) correction on ONT raw reads (left) and PacBio raw reads (right) in relation to the reference frequencies. The top 50 six-mers with the greatest change in frequency are displayed.

As expected, the occurrence of k-mers in corrected reads was much more strongly correlated to the k-mer composition of the reference than without correction. The correction by proovread using Illumina data improved k-mer occurrences similarly well in both datasets, slightly better in ONT than in PacBio reads (Figure 10). This slight superiority of ONT data was mainly due to remaining over-represented homopolymers in PacBio data. The Canu correction in PacBio data resulted in a larger number of correct k-mers than in ONT data. For ONT reads, many six-mers occurring in the reference genome and also being present in the raw data showed reduced numbers after Canu correction; there was a tendency that six-mers which were already slightly under-represented in the raw reads, were even more depleted by Canu correction (Figure 11). Only a few of these under-represented six-mers were changed by the Canu correction towards a correct representation, this was observed for both Canu-supported alignment methods (MHAP or minimap2) and for both unpatched and patched input reads. In numbers, 61 of 4096 ONT six-mers (1.48%) occurred more than 3*stddev further off the diagonal ( = perfect representation) after the Canu correction step.

## DISCUSSION

With the advancement of extended read lengths in third-generation sequencing data, high error rates are the main challenge when working with long-read data. We observed total error rates around 13% in PacBio and ONT raw data in agreement with previous studies. When assessing substitution rates and insertion/deletion rates separately we obtained slightly different results depending on the alignment tool. Also, the total number of aligned reads and the length of the longest alignment were dependent on the choice of the alignment method. Considering average values of all methods, we found error rates around 4% (±0.5%) for each type of errors (substitutions, insertions, deletions) in ONT data, whereas PacBio data showed low substitution rates (1.7%), medium deletion rates (3.2%) and high insertion rates (8.0%). Another difference between ONT data and PacBio data was the type of substitutions: transitions (A↔G, C↔T) were the most prominent substitutions in ONT data, whereas transversions (A↔C, G↔T) were elevated in PacBio data. Homopolymers were among the most over-represented six-mers in both datasets. In PacBio data, the four homopolymers appeared at positions 1 (polyT), 2 (polyA), 5 (polyG), 6 (polyC) in the list of the most over-represented six-mers. In ONT data, polyA and polyT were at the top two positions, while polyG appeared at position 13, and the polyC six-mer appeared far down in the list at position 974, i.e. was the only homopolymer that was close to perfect representation. The sequence composition of over-represented six-mers also differed clearly between ONT and PacBio data: C and G were more often in over-represented ONT six-mers than T and A, whereas T was the dominating nucleotide in over-represented PacBio six-mers.

After applying patching (MARVEL), consensus correction (Canu, MARVEL), and short-read -assisted correction (proovread) in various combinations we found that (i) patched input data achieved lower error rates than unpatched input data, (ii) long-read consensus correction achieved lower error rates than short-read-assisted correction for PacBio data, (iii) short-read-assisted correction achieved lower error rates than long-read consensus correction for ONT data, (iv) in PacBio data, Canu consensus correction after MARVEL patching achieved lower error rates than MARVEL consensus correction after MARVEL patching, whereby Canu using minimap2 achieved lower error rates than Canu using MHAP as alignment method.

Long-read consensus correction methods applied on PacBio data resulted in error rates below 1% no matter if patched or unpatched input data were used. For ONT data, error rates below 1% could only be achieved by using additional Illumina data with proovread and only for patched input data.

The lowest error rate for ONT data was 0.45% using proovread after MARVEL patching. The lowest error rate for PacBio data was 0.42% using Canu with minimap2 after MARVEL patching.

The post-correction error rates achieved for PacBio data were similar to the ones obtained after self-correction using PacBio high-fidelity data (34). Contrasting to PacBio where the creation of a circular consensus sequence nowadays is an option even for genomic templates, no such possibility exists for ONT sequencing reads.

Taken together, we provide an evaluation of the outcome of different correction procedures applied on noisy long-read data as generated by PacBio and ONT sequencing platforms, respectively. In order to obtain most benefit from long sequencing reads, corrected reads are of high importance. Our study provides valuable insights in the characteristics of uncorrected and corrected sequencing reads and shows how to achieve lowest error rates using different correction methods.

## Supplementary Material

lqaa037_Supplemental_File

## Notes

Present address: Philipp Peters, Next-Generation Sequencing Facility, Vienna BioCenter Core Facilities, (VBCF), Dr.-Bohr-Gasse 3, 1030 Vienna, Austria.
