# Supplementary material for: Benchmarking of long-read correction methods
Source: NAR Genom Bioinform. 2020 May 25;2(2):lqaa037. doi: 10.1093/nargab/lqaa037 (PMC7671305; doi:10.1093/nargab/lqaa037)
Supplement: lqaa037_Supplemental_File [file lqaa037_supplemental_file.pdf]

Benchmarking of long-read correction methods

Juliane C. Dohm, Philipp Peters, Nancy Stralis-Pavese, Heinz Himmelbauer

Supplementary Figures

Supplementary Figure S1: Zoomed comparison of occurrences of six-mers (a), seven-mers (b), eight-mers (c) in the reference genomes and the raw read sets of ONT (left) and PacBio (right). The diagonal blue line stands for perfect representation. The two red lines show the 3-fold standard deviation.

a)

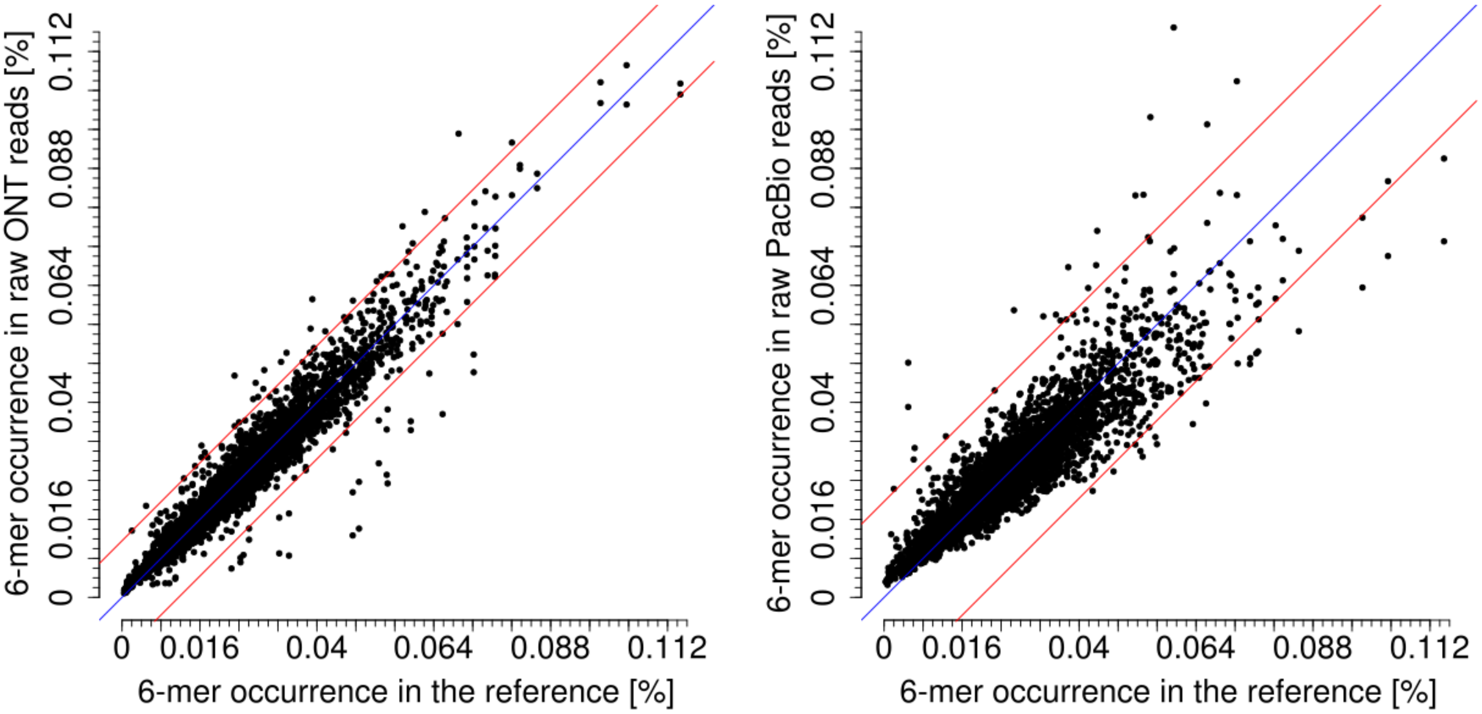

b)

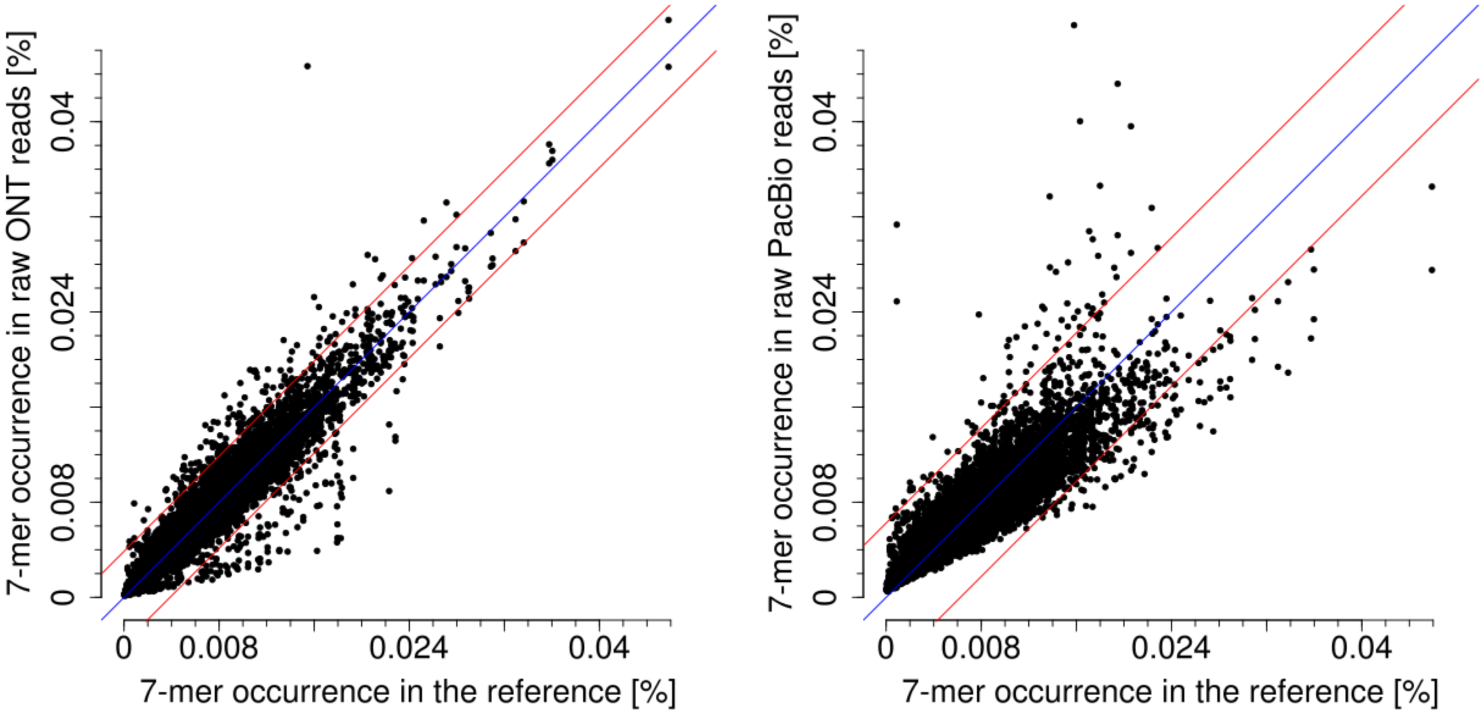
7-mer occurrence in raw ONT reads [%]
0
0.008
0.024
0.04
7-mer occurrence in the reference [%]
7-mer occurrence in raw PacBio reads [%]
7-mer occurrence in the reference [%]

c)

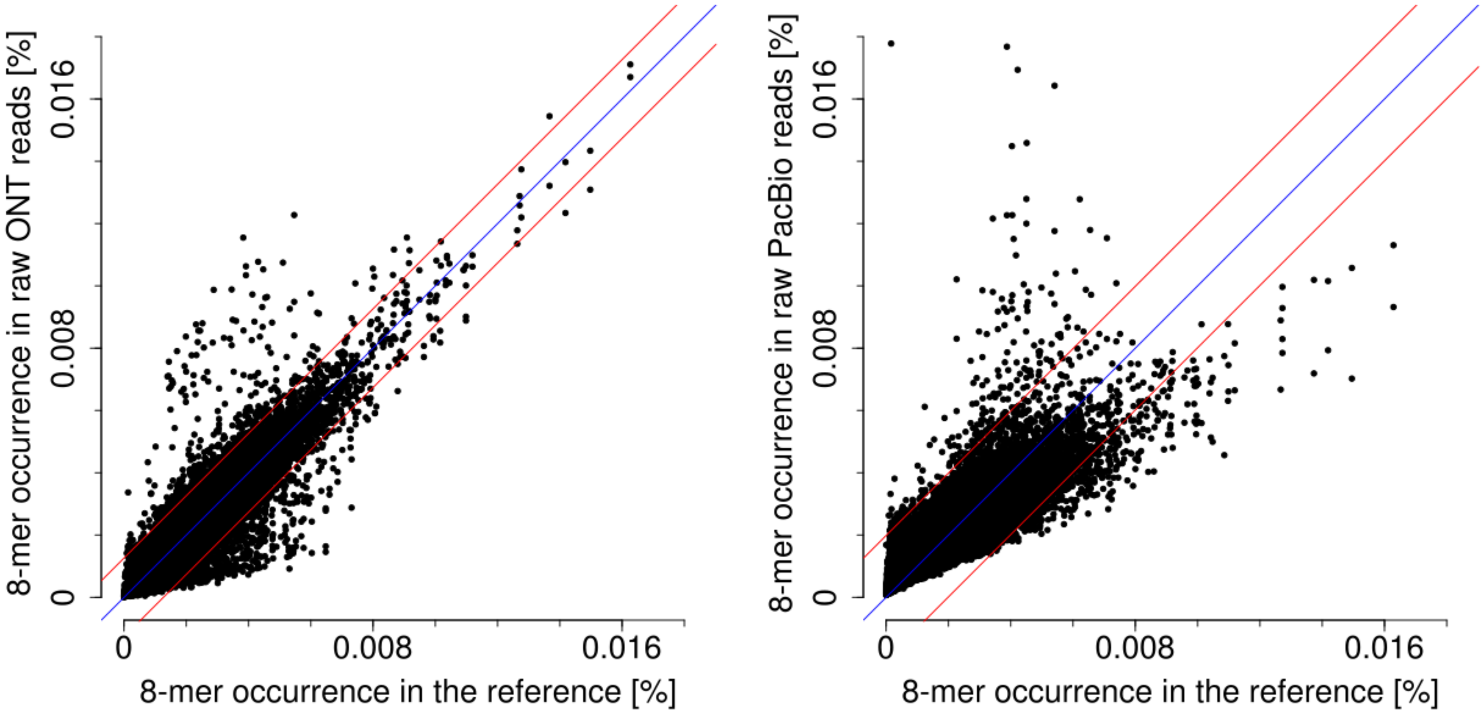
8-mer occurrence in raw ONT reads [%]
0
0.008
0.016
8-mer occurrence in the reference [%]
8-mer occurrence in raw PacBio reads [%]
8-mer occurrence in the reference [%]

Supplementary Figure S2: Frequencies of six-mers in raw reads and patched reads of ONT (left) and PacBio (right) data. Blue line: perfect representation, red lines: 3-fold standard deviation. The six-mers furthest away from the diagonal represent the homopolymers.

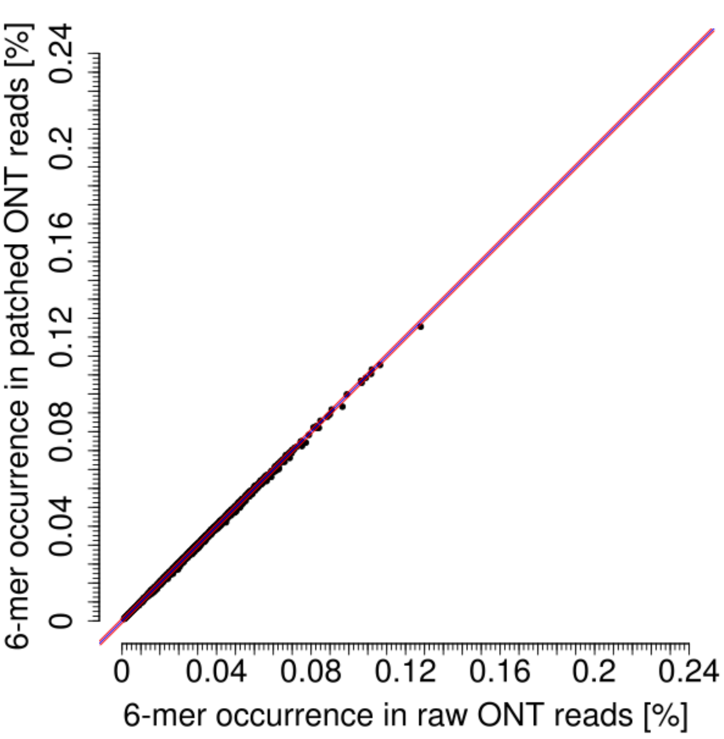

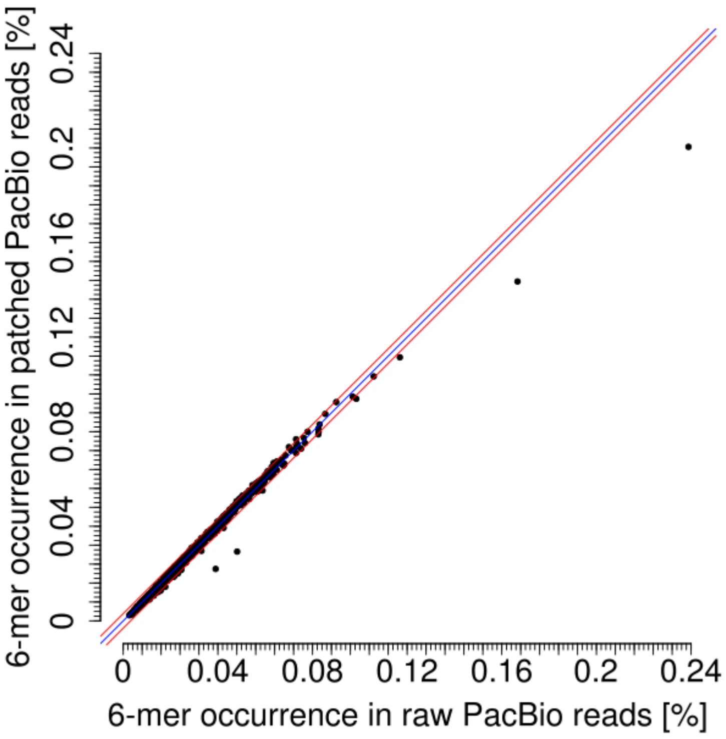

Supplementary Table

Supplementary Table S1: The 30 most over- and underrepresented six-mers in the raw read datasets compared to the reference.

	ONT		PacBio	
	6mer	difference in rate	6mer	difference in rate
overrepresented	TTTTTT	0.0573	TTTTTT	0.1699
	AAAAAA	0.0260	AAAAAA	0.0977
	GCACGG	0.0224	GTTTTT	0.0575
	CGGGCG	0.0221	TTTTTG	0.0439
	CGGCGG	0.0186	GGGGGG	0.0432
	CACGGC	0.0174	CCCCCC	0.0341
	CAGCGG	0.0169	TTTTTC	0.0335
	GGGCGG	0.0166	TTTTGG	0.0323
	CGGTGG	0.0164	TGTTTT	0.0315
	CCGGGC	0.0164	GGTTTT	0.0309
	GGCGGG	0.0157	ATTTTT	0.0308
	CACGGG	0.0148	TTTTAA	0.0300
	GGGGGG	0.0138	TTTTTA	0.0294
	TCCCGC	0.0138	TTGTTT	0.0250
	CCACGG	0.0138	AATTTT	0.0247
	GTTTTC	0.0135	GCCGGC	0.0244
	GCGGCG	0.0133	AGTTTT	0.0227
	CACGGT	0.0132	CGGCCG	0.0222
	GCAGCG	0.0130	GTTTTG	0.0216
	GTTTTG	0.0129	TTTAAA	0.0206
	GGTGGG	0.0127	TTTGGG	0.0204
	GCGGTG	0.0126	GGCGCC	0.0203
	GCGCGC	0.0126	GGGTTT	0.0198
	CGGGGC	0.0124	CGTTTT	0.0197
	GCGTTT	0.0121	TTTGGC	0.0196
	TGGGTG	0.0120	TTTTGT	0.0192
	CACGGA	0.0119	CTTTTT	0.0185
	TCACGG	0.0119	TTTTCC	0.0181
	CGTTTT	0.0119	TTTTCG	0.0176
	CGCGCG	0.0118	GCTTGG	0.0173

	ONT		PacBio	
	6mer	difference in rate	6mer	difference in rate
underrepresented	GCCTGG	-0.0347	CGCCAG	-0.0418
	CCTGGC	-0.0345	CCAGCG	-0.0346
	CAAAAA	-0.0311	GCCAGC	-0.0334
	AAAAAG	-0.0292	CCAGCA	-0.0305
	AAAAAT	-0.0282	CACCAG	-0.0278
	GAAAAA	-0.0260	CCGCCA	-0.0272
	CCAGGC	-0.0258	TGCCAG	-0.0263
	ACCTGG	-0.0257	TCGCCA	-0.0263
	TAAAAA	-0.0252	ACCAGC	-0.0263
	GCCAGG	-0.0250	ATCGCC	-0.0261
	AAAAAC	-0.0250	CTGGCG	-0.0248
	CCTGGT	-0.0233	TCCAGC	-0.0246
	GTTTTT	-0.0231	ATCACC	-0.0246
	TTTTTC	-0.0224	CCACCA	-0.0242
	CTTTTT	-0.0199	ACCGCC	-0.0228
	CGCCTG	-0.0172	TCACCA	-0.0226
	CCTGGG	-0.0171	CACCAC	-0.0212
	CCAGGT	-0.0171	ACCACC	-0.0210
	CCCTGG	-0.0166	CGCTGG	-0.0202
	TTTTTA	-0.0164	TCACCG	-0.0202
	CCTGGA	-0.0162	CAGCAG	-0.0198
	CCCAGG	-0.0162	GCCAGT	-0.0196
	TTTTTG	-0.0160	TTACCG	-0.0195
	ACCAGG	-0.0159	GCCGCC	-0.0193
	TCCTGG	-0.0143	CACCGC	-0.0192
	AACCTG	-0.0129	CGCCGC	-0.0190
	GCAAAA	-0.0129	TCATCA	-0.0190
	TGCCTG	-0.0122	CGCCAT	-0.0187
	CCAGGA	-0.0120	TTCACC	-0.0186
	ATTTTT	-0.0117	TTCCAG	-0.0184
